# Enhanced Delivery of 4-Thioureidoiminomethylpyridinium Perchlorate in Tuberculosis Models with IgG Functionalized Poly(Lactic Acid)-Based Particles

**DOI:** 10.3390/pharmaceutics11010002

**Published:** 2018-12-21

**Authors:** Leonid Churilov, Viktor Korzhikov-Vlakh, Ekaterina Sinitsyna, Dmitry Polyakov, Oleg Darashkevich, Mikhail Poida, Galina Platonova, Tatiana Vinogradova, Vladimir Utekhin, Natalia Zabolotnykh, Vsevolod Zinserling, Peter Yablonsky, Arto Urtti, Tatiana Tennikova

**Affiliations:** 1 Faculty of Medicine, Saint Petersburg State University, 7/9 Universitetskaya Embankment, 199034 St. Petersburg, Russia; elpach@mail.ru (L.C.); poidan@mail.ru (M.P.); utekhin44@mail.ru (V.U.); v.zinserling@spbu.ru (V.Z.); glhirurgb2@mail.ru (P.Y.); 2 Institute of Chemistry, Saint Petersburg State University, 7/9 Universitetskaya Embankment, 199034 St. Petersburg, Russia; v.korzhikov-vlakh@spbu.ru (V.K.-V.); kat_sinitsyna@mail.ru (E.S.); ravendoctor@mail.ru (D.P.); arto.urtti@helsinki.fi (A.U.); 3 Institute of Macromolecular Compounds, Russian Academy of Sciences, Bolshoi pr. V.O. 31, 199004 St. Petersburg, Russia; gplaton@mail.ru; 4 Republican Center for Innovative and Technical Creativity, Slavinskogo str. 12, 220086 Minsk, Belarus; centre@rcitt.by; 5 St. Petersburg Research Institute of Phthisiopulmonology, Polytechnical str. 32, 194064, St. Petersburg, Russia; vinogradova@spbniif.ru (T.V.); zabol-natal@yandex.ru (N.Z.)

**Keywords:** tuberculosis, polymeric nanoparticles, poly(lactide), 4-thioureidoiminomethylpyridinium perchlorate (perchlozone), macrophage, camel mini-antibodies, opsonization, drug delivery

## Abstract

The compound 4-thioureidoiminomethylpyridinium perchlorate (perchlozone^©^) is a novel anti-tuberculosis drug that is active in multiple drug resistance cases, but the compound is hepatotoxic. To decrease the systemic load and to achieve targeting, we encapsulated the drug into poly(lactic acid)-based micro- (1100 nm) and nanoparticles (170 nm) that were modified with single-chain camel immunoglobulin G (IgG) for targeting. Both micro- and nanoparticles formed stable suspensions in saline solution at particle concentrations of 10–50 mg/mL. The formulations were injected intraperitoneally and intravenously into the mice with experimental tuberculosis. The survival of control animals was compared to that of mice which were treated with daily oral drug solution, single intraperitoneal administration of drug-loaded particles, and those treated both intravenously and intraperitoneally by drug-loaded particles modified with polyclonal camel IgGs. The distribution of particles in the organs of mice was analyzed with immunofluorescence and liquid chromatography/mass spectrometry. Morphological changes related to tuberculosis and drug toxicity were registered. Phagocytic macrophages internalized particles and transported them to the foci of tuberculosis in inner organs. Nanoparticle-based drug formulations, especially those with IgG, resulted in better survival and lower degree of lung manifestations than the other modes of treatment.

## 1. Introduction

Tuberculosis (TB) represents an international health priority and burden, affecting about 10.4 million people annually [1,2]. Human immunodeficiency virus (HIV)/TB co-infection and socioeconomic difficulties make treatment of tuberculosis difficult in many developing countries. Currently, about 1.7 million TB patients die annually, and migration of people tends to spread TB further. Multiple drug resistance of *Mycobacterium tuberculosis* (MDR-TB) is a major concern in current tuberculosis therapy. According to World Health Organization (WHO) estimates, there are about 490,000 new cases of MDR-TB annually (http://www.who.int/news-room/fact-sheets/detail/tuberculosis). Thus, effective strategies to overcome this problem are urgently needed [2,3,4], but the development of new effective drugs is slow. 

Recently, a new anti-TB drug, 4-thioureidoiminomethyl pyridinium perchlorate (perchlozone), was introduced and registered in several countries by Pharmsyntez (Irkutsk, Russia). The compound is effective against MDR-TB, but it may cause hepatic toxicity [5,6,7]. Thus, reduction of toxicity-related drug exposure is needed, while maintaining drug efficacy. 

In principle, anti-TB medications could be improved by using carriers that deliver the drug to the infection foci, thereby reducing the required drug doses and potentially decreasing drug toxicity. Previously, anti-microbial compounds were encapsulated into biodegradable micro- and nanoparticles of different polyesters [8,9,10]. Also, some anti-TB drugs were loaded into poly(lactic-*co*-glycolic acid) (PLGA)-based microparticles and administered via pulmonary route for treatment of experimental TB in mice [11]. The pulmonary administration of the particles resulted in drug internalization into the alveolar macrophages [12,13,14]. Likewise, intravenous (i.v.) delivery in PLGA nanoparticles enhanced rifampicin disposition to the lung [15], but the mechanisms of particle transfer to the target tissue remained unclear. 

Interestingly, the macrophages and their descendants harbor *Mycobacteria* and accumulate in the infectious foci [16]. Therefore, targeting of anti-TB compounds to these cells may be an effective strategy to improve therapeutic index of these drugs. Rifampicin and ofloxacine were delivered into alveolar macrophages with inhaled liposomes, hyaluronic acid microspheres, poly(d,l-lactic-*co*-glycolic) acid and poly(ester-*co*-amides) [17,18,19,20]. The targeted delivery of anti-TB drugs into alveolar macrophages was also achieved by conjugation of phagocyte receptor specific tuftsin peptide or other targeting moieties to the particle surfaces [21,22,23]. 

Such drug delivery systems combine features of polymeric particles and intrinsic transfer properties of macrophages, constituting a biohybrid approach [24,25]. Polyester-based micro- and nanoparticles may be useful in this context, because they can entrap drugs and protect the drug inside the phagocytes. 

Phagocytosis of perchlozone-loaded particles by macrophages and subsequent localization in the infected foci might result in improved efficacy and safety of the drug [23]. In this study, we loaded perchlozone into the nano- and microparticles of poly(d,l-lactic acid) for targeting the drug via macrophage-mediated chemotaxis into the TB-infected foci in mice. This approach resulted in targeted drug delivery and effective treatment at reduced dosing levels of perchlozone. 

## 2. Materials and Methods

### 2.1. Materials and Instruments

All commercially available chemicals were purchased from Fluka (Buchs, Switzerland) and Sigma-Aldrich (Darmstadt, Germany) and used without further purification. The poly(d,l-lactic acid) (PLA, molecular weight (M_W_) 24,000) for particle preparation was synthesized and characterized according to a previously published procedure [26]. Spin columns (molecular weight cut-off (MWCO) 3000 and 100,000; VIVASCIENCE, Sartorius Group, Göttingen, Germany) were used for dialysis, particle purification and separation. Papain was purchased from Sigma-Aldrich (Germany). Fluorescein isothiocyanate (FITC)-labeled zymosan was taken from Molecular Probes (Thermo Fisher Scientific, Waltham, MA, USA).

Perchlozone was extracted from commercially available tablets (Pharmasyntez OJSC, Russia) as follows: 2 tablets, containing 400 mg of perchlozone each, were placed into a glass tube, and 5 mL of distilled water was added. The tube was stirred with a vortex to break down the particles and heated up to 80 °C for one hour. Then, the deep-yellow solution was separated by filtration and, while cooling down, perchlozone crystallization was observed. The yield was 680 mg of active component. The compound melted at 238–240 °C. The FTIR spectrum of substance was as follows (KBr), ν, cm^−1^: 3420 (NH_2_); 3310 (NH); 1630 (C=N); 1495 (C=C, ring); 1355 (C–N); 1120 (C=S); 1040 (ClO_4_^−^). ^1^H NMR spectrum of substance (dimethyl sulfoxide (DMSO-*d*_6_)), δ, ppm: 8.11 (1H, =CH, *s*), 8.43 (2H, H-3.5, *d*, 3*J* = 6.5 Hz), 8.54 (1H, NH_2_, *bs*), 8.66 (1H, NH_2_, *bs*), 8.85 (2H, H-2,6, *d*, 3*J* = 6.5 Hz), 12.11 (1Н, NH, *bs*). The ultraviolet (UV) spectrum of substance in water possesses two maxima at 232 and 318 nm. The purity of obtained drug was more than 98% according to HPLC (Russian Pharmaceutical Standard No000037-241220). 

### 2.2. Animals

#### Animals

Mice. All pathophysiological experiments were organized and maintained on principles of humane care of animals with consent and approval of the bioethical review committee of the Research Institute of Pthisiopulmonology (Statement #48.1). In total, 152 outbred male albino mice (Rappolovo nursery, St. Petersburg, Russia) weighing 19 ± 1 g were used. The eight-week-old [27] outbred mice were chosen to mimic the large genetic variability [28] of human patients. Animals prior to the study were quarantined for 14 days and monitored daily by visual clinical inspection. Only clinically healthy mice with positive body mass dynamics were included in the experiments. They were given a standard diet fortified in protein and vitamins (Furage Ltd., St. Petersburg, Russia), recommended by Russian Health Ministry edict #1179 for TB-related animal experimentation, plus water ad libitum. The mice were kept at the vivarium of Research Institute of Phthisiopulmonology under standard conditions in full accordance with the “Rules of an establishment, equipment, and maintenance of experimental biological clinics”, approved by Russian Federation (RF) State standard R53434-2009. The light conditions were 12 h light/12 h darkness, air temperature was maintained between 23 and 25 °C, and relative air humidity was 50–70%. Air exchange was carried out by inflow/outflow ventilation, with daily air sterilization by UV light. The mice were housed in groups of 9–15 animals in polycarbonate cages, with a floor area of at least 56.3 cm^2^ per animal, covered by pre-disinfected sawdust. 

Camel. A donor of camel immunoglobulin (Ig) was a healthy double-humped Bactrian female camel of Kalmyk breed, age three years, body mass 450 kg, from the stable of St. Petersburg State Academy of Veterinary Medicine (SPbSAVM). Feeding was carried out on a complete diet recommended by SPbSAVM, and maintenance was in the stall.

All experiments were carried out in accordance with European Union (EU) Directive 2010/63/EU for animal experimentation, under conditions of humane care of experimental animals, with due euthanasia of survived mice under ether narcosis. 

### 2.3. Methods

#### 2.3.1. Preparation of Perchlozone-Loaded Polymeric Micro- and Nanoparticles

Microparticles. The double emulsion evaporation method [26] was applied for perchlozone encapsulation into the PLA microparticles. The optimized procedure was as follows: a solution of 300 mg of the drug and 50 mg of lecithin in 1.5 mL of 0.01 M sodium phosphate buffer, pH 7.4, was prepared. This solution was emulsified in 15 mL of organic phase (10 mL of dichloromethane, 5 mL of acetone, 1 g of PLA, 0.5 g of lecithin) using an ultrasound homogenizer Sonopuls HD2070 (Bandelin, Berlin, Germany) and magnetic stirrer MR Hei-Mix S (Heidolph, Schwabach, Germany). After 2 min of emulsification, the resulting emulsion was added during 3 min to 45 mL of aqueous phase (1 wt.% poly(*N*-vinylpyrrolidone), 0.5 wt.% sodium dodecyl sulfate, 0.5 wt.% Lutrol F68) under stirring at 1000 rpm and simultaneous sonication to form a double emulsion. Thereafter, the organic solvent was removed from the mixture at reduced pressure with application of Hei-VAP Precision ML/G3B rotary evaporator (Heidolph), one hour at 100 mbar and one hour at 50 mbar. The microparticle suspension was centrifuged at 4000× *g* for 10 min with application of Sigma 2-16 P (Sigma, Darmstadt, Germany) centrifuge. The separated particles were washed four times with distilled water and resuspended in 0.1 M sodium phosphate-buffered saline, pH 7.4, in which they were stored at 4 °C until application and resuspended immediately before injection.

Nanoparticles. The nanoparticles were obtained by nanoprecipitation similar to an earlier described procedure [29]. A solution of 100 mg of poly(lactic acid) in 20 mL of drug solution in acetonitrile was slowly added to 100 mL of water. The mixture was stirred for two days in an opened flask to eliminate the organic solvent. The particles were purified from free drug via dialysis against 0.1 M phosphate-buffered saline, pH 7.4, using Vivaspin 20 columns (Sartorius, Göttingen, Germany, MWCO 100,000) and the abovementioned centrifuge. 

For biological imaging purposes, both nano- and microparticles were labeled with platinum(II)-coproporphyrin (PtCP). This was done by addition of 50 μL of PtCP (1 mg/mL in ethanol) to perchlozone solution just before preparation of particles. 

#### 2.3.2. Particle Characterization

Particle size, zeta potential, and size distribution. The particle size, zeta potential, and size distribution of freshly prepared particles were determined with dynamic light scattering (DLS) with application of a Zetasizer Nano ZS (Malvern, Enigma Business Park, UK) instrument. The mean size of lyophilized particles was estimated from scanning electron microscope images obtained with a Zeiss Supra 55VP (Basel, Switzerland) instrument and Fiji ImageJ free software (version 1.51j, Laboratory for Optical and Computational Instrumentation (LOCI) at the University of Wisconsin-Madison, Madison, WI, USA, 2017). 

Drug loading and encapsulation efficiency. Fourier-transform infrared (FTIR) and X-ray diffraction (XRD) measurements were carried out with a Nicolet 8700 spectrometer (Thermo Fisher Scientific) and D2 Phaser diffractometer (Bruker, Billerica, MA, USA). The samples of particle suspensions (0.3 mL) were washed three times with distilled water and lyophilized with application of FreeZone 1L Benchtop Freeze Dry System (Labconco, Kanzas City, MO, USA). Dried particles were weighed and dissolved in acetone to evaluate the quantity of perchlozone by measuring the adsorption of the light at λ = 380 nm (molar extinction coefficient—5400) with a UV mini-1240 spectrophotometer (Shimadzu, Tokyo, Japan). The drug loading (mg) and encapsulation efficiency (%) were calculated as follows:

DL = Q_particles_ × m_particles_, (1)

EE (%) = DL × 100/DL_0_, (2)

where Q_particles_ is the quantity of drug entrapped into 1 mg of particles, m_particles_ is the total weight of the particles (mg), and DL_0_ is the theoretical drug loading, calculated as DL_0_ = С_0_V_0_/m_particles_, where С_0_ is initial concentration of the drug solution and V_0_ is solution volume. 

Particle degradation. The degradation of the particles was analyzed by measuring their weight loss as described earlier [26]. Dry particles (100 mg) were placed into Eppendorf^®^ microtubes and mixed with 0.01 M phosphate-buffered saline (PBS, pH 4.5) with 0.1% SDS and 0.5 mg/mL papain. This medium was used to mimic lysosomal acidic pH in the macrophages. At certain time periods, the particles were centrifuged, washed with water, freeze-dried, and weighed with an Ohaus AV313 Adventurer Pro Digital Balance (Moscow, Russia) to evaluate the weight loss of the particles. The weight loss (%) was calculated as follows:

PWL (%) = [(W_0_ − W_t_)/W_0_] × 100%, (3)

where W_0_ is the initial weight of the particles, and W_t_ is their weight at certain time (t) in the medium. 

Drug release tests. The release study was conducted in a medium that mimics intra-lysosomal conditions in macrophages. The medium contained 0.01 M phosphate-buffered saline (pH 4.5), 0.1% SDS and 0.5 mg/mL papain. At defined times during the experiment, the particle suspensions were centrifuged (microparticles 5000× *g* for 5 min; nanoparticles 12,000× *g* for 5 min). Then, 200-µL aliquots of supernatant were withdrawn and the concentration of released perchlozone was determined spectrophotometrically (NanoDrop 2000, ThermoFisher Scientific) at λ = 318 nm. Cumulative drug release (%) vs. time curves were generated. 

The release data were approximated with application of Higuchi (Equation (4)) and Korsmeyer–Peppas (Equation (5)) release models [30,31]:

Q = K_H_t^1/2^. (4)

Log(M_t_/M_∞_) = Logk + nLogt. (5)

#### 2.3.3. Camel IgG Fractionation and Attachment to the Particles

Camel blood was collected from the jugular vein into a hemotransfusion container (Kompoplast 300/300, Syntez OJSC, St. Petersburg, Russia) with sodium citrate. Thereafter, plasma and cells were isolated by centrifugation as described elsewhere [32]. Concentrations of different IgG classes in serum were estimated by their sedimentation at their isoelectric point: IgA = 6.89 g/L; IgM = 0.2 g/L; IgG_1_ = 10.7 g/L; IgG_2_ = 0.71 g/L. Determination of antimycobacterial Ig in camel blood serum was performed by ELISA method using the kits in "SD Bioline TB WB" (Standard Diagnostics Inc, Borahagal-ro, South Korea) by competition with antimycobacterial standard-calibrated human immunoglobulin samples. The activity was compared to that in uninfected healthy human sera donors and TB patients with various forms of TB (from Biobank of Saint Petersburg Research Institute of Phthisiopulmonology, St. Petersburg, Russia). The approximate level of camel anti-TB IgG (391 ± 36 units (U)) was estimated to be comparable to that of patient with fresh exacerbation of TB (1040 U), much higher than in an uninfected healthy donor (<50 U) and significantly higher than in non-exacerbated patients with fibro-cavernous (271 U) or infiltrative (209 U) pulmonary TB. A lymphocyte blast transformation test with 20 mg/mL tuberculin (PAO Farmstandart–Biolek, Kharkov, Ukraine) was positive in 2.2% of camel lymphocytes. Antimycobacterial activity was preserved in the serum for more than 80% after a single freeze thawing. We assume that it resulted from natural immunization. 

Camel IgGs were isolated from blood plasma by affinity chromatography on a protein A Bio-RAD column (Bio-Scale TM Mini Affi-Prep® Protein A Cartridge, Bio-Rad Laboratories Inc., Moscow, Russia) in 0.01 М sodium phosphate-buffered saline, pH 7.4. Then, 1% acetic acid solution, pH 3.0, was applied for IgG detachment and dry Tris buffer was used for its neutralization.

The surface of both micro- and nanoparticles was carboxylated by treatment with 0.01 M sodium hydroxide for 30 min. The carboxylic groups were then activated with excess of 1-hydroxybenzothriazole/1-ethyl-3-(3-dimethylaminopropyl)carbodiimide mixture in 0.01 M 2-(*N*-morpholino)ethanesulfonic acid (MES) buffer solution (pH 5.5). The camel IgG was coupled to the particles in 0.01 M sodium borate buffer at pH 8.8. The quantity of coupled IgG (5 μg/mg of particles) was estimated photometrically by analyzing supernatant solution at 280 nm. 

#### 2.3.4. Ex Vivo Determination of Particle Phagocytosis by Peritoneal Macrophages

The phagocytosis of cells in the fluid was proved with FITC-labeled zymosan according to a previously published procedure [33]. The morphology of phagocytes was estimated with optical microscopy, and they had all peculiarities of macrophages. 

The micro- and nanoparticles were prepared as described above and covalently modified by Cy3-NHS-labeled bovine serum albumin (BSA) and camel IgG. The Cy3 was introduced for particle visualization by confocal laser microscopy. 

In these experiments, 25 mice were used as five groups with five animals in each: (1) control group; (2) group treated with PLA–BSA–Cy3 microparticles; (3) group treated with PLA–BSA–Cy3 nanoparticles; (4) group treated with PLA–camel IgG–Cy3 microparticles; (5) group treated with PLA–camel IgG–Cy3 nanoparticles. Particle suspensions (0.5 mL, 10 wt.%) in saline were injected intraperitoneally (groups 2–5). After 20 min, the animals were euthanized and 5 mL of saline solution was administered intraperitoneally (i.p.) to wash out the remaining particles. Fluid (2.5–3.0 mL) was withdrawn from the peritoneum of each mouse, and 1 mL of the retrieved liquid was placed on the coverslips in Petri dishes and incubated for 1 hour at 5% CO_2_ and 37 °C. Then, the dish content was washed with saline solution (at 30 °C) and fixed with 4% paraformaldehyde for 40 min in the darkness. Then, the samples were washed with saline three times for 20 min and stored in the freezer (4 °C) until analyzed [34].

The samples were stained in the dark with 4′,6-diamidino-2-phenylindole (DAPI; 0.36 µM in PBS) for 5 min at room temperature (25 °C) and washed twice with 0.01 M PBS, pH 7.4. The coverslips were attached to the microscope slides with Dako mounting medium (Agilent Technologies, Santa Clara, CA, USA) equipped with Plan-Apochromat 20×/0.8 M27 and C-Apochromat 40×/1.20 W CorrUV-VIS-IRM27 objectives. For excitation of Cy3 and DAPI fluorescence, 561-nm and 405-nm lasers were applied, respectively. The fluorescence of Cy3 was detected with a 570–613-nm filter, and DAPI was imaged with a 420–480-nm filter. The images in transmitted light were obtained using a differential interference contrast technique. The images were converted into JPEG files by application of software supplemented to the confocal microscope. 

The phagocytosis efficacy (%) was calculated as the percentage of the cells with particles compared to the total number of cells. 

#### 2.3.5. Detection of Particles in Different Organs 

To check the distribution of particles, we studied the fluorescence of encapsulated PtCP in the tissues of different organs. Laparotomy was performed to mice under ether narcosis on the first, second, and seventh day after i.p. injection of particles. Fluorescence of inner organs from perished or euthanized mice was registered. Paraffin-embedded tissue slices were studied using a laser fluorometric scanner Diagem (Immunoscreen Inc., Moscow, Russia) [35] at excitation length 350 nm and detection at 662 nm with a delay of 20 µs. Quantitative estimation of fluorescence intensity was performed by integration of a signal over total slice square. To visualize the PtCP-loaded particles fluorescence in the lung, the fluoromicroscope attachment (Immunoscreen Inc.) was used.

#### 2.3.6. Investigation of Perchlozone Distribution 

Perchlozone analysis. Liquid chromatography/mass spectrometry (LC–MS) analyses were performed with a Shimadzu 8030 (Kyoto, Japan) chromatograph, supplied with degasser, binary pump, autosampler, thermostate, multi-wavelength UV–visible light (Vis) detector and MS-detector QQQ. The separation was carried out with application of a Phenomenex column (Luna C18 5 μm, 150 × 2.1 mm). As the mobile phase in liquid chromatography, we used a mixture of acetonitrile (ACN) and 0.1% trifluoroacetic acid (TFA; 85:15) (pH = 2.6). The flow rate was 1 mL/min and temperature was 40 °C. The injected sample volume was 2 μL. Ionization in mass spectrometry was carried out by means of electrospray at atmospheric pressure: voltage on the capillary—1900 V, drying nitrogen velocity—13.3 mL/min, spraying nitrogen velocity—2.7 mL/min, and drying gas temperature—250 °C. The detection was carried out in a positive mode of analysis by the multiple reaction monitoring (MRM) method by taking the 183.2 *m*/*z* peak as a molecular ion. Transitions were 183.2–142.2 at 20 eV and 183.2–113.2 at 20 eV. The retention time of the perchlozone was 1.39 min and total analysis time was five minutes.

Calibration curve. Perchlozone was isolated from a powder substance. A weighed portion of the powder (it was assumed that the perchlozone content was as in tablet, equal to 67%) of 5 mg was dissolved in 1 mL of MeOH, stirred with a vortex, and placed into an ultrasonic bath for 15 min. Then, the suspension was centrifuged for 30 min at 20 °C at a speed of 13,000 rpm. Then, the solution was filtered through a filter having a pore diameter of 0.22 µm, and the solution was dried in a vacuum at 45 °C. The precipitate was stored at −70 °C before analysis, dissolved in 110 µL of ACN:0.1% TFA, pH = 2.6 at a ratio of 10:1. Next, standard solutions were prepared by dissolving the initial solution by eluent. To calibrate the instrument and validate the procedure, solutions with a concentration of 0.07, 0.33, 0.67, 3.33, and 13.33 µg/mL were used. The calibration chart had values of *R* = 0.998, and relative standard deviation (RSD%) = 27%.

Slice analysis. One-third of the slice was mechanically detached from the paraffin and transferred to the Eppendorf tube. Then, 1 mL of 80% methanol was added, and the sample was placed into a homogenizer and heated to 70 °C for 45 min. Then, the tube was transferred to the ice for 10 min and centrifuged at 4 °C at 13,000 rpm. The supernatant was taken out and lyophilized. The dried powder was dissolved in 100 μL of eluent and mixed with a vortex, centrifuged (10 min at 13,000 rpm), and diluted 100-fold with eluent. The solution was filtered and transferred to the vial for detection of perchlozone concentration using LC–MS.

#### 2.3.7. In Vivo Experiments with Tuberculosis Mice

The mice were separated into seven groups (Table 1). The remaining non-infected mice were used for model experiments to explore dynamics of particle distribution after single i.p. injection. 

Mycobacteria and inoculation procedure. MDR-TB, strain H37R_v_ TBC # 1/47, sensitive to existing anti-TB drugs, was procured from a culture collection of the Institute of Hygiene and Epidemiology (Prague, Czech Republic). It was maintained on Lőwenstein–Jensen medium through routine bimonthly passage. To inoculate infection, mycobacterial growth of the second generation (21 days old) maintained on solid Lőwenstein–Jensen medium was harvested (according to the guide on the holding of preclinical research of drugs, Ministry of Healthcare and Social Development of the Russian Federation, similar to that published in Reference [36]). It was weighed, and a 5 mg/mL suspension was prepared by homogenizing the culture in sterile saline with glass beads as described below. The bacterial mass collected from the dense medium was placed in a dry sterile glass test tube with 8–10 glass beads with a diameter of 3 mm, and triturated with a vortex shaker for 15–20 s. Then, three drops of saline were added and triturated further. Then, 5–6 mL of saline was added and the stirring procedure was repeated. The suspension was then transferred to a centrifuge tube and the clamps were precipitated at 1000 rpm for 10 min. The upper part of the supernatant was carefully withdrawn, avoiding capture of the clamps in the lower layers, and transferred to a polystyrene tube for turbidity measurements. The suspension was adjusted to 1.5 units according to McFarland 5 × 10^8^ cells/mL using a DENSI-LA-METER II (Erba Lachema s.r.o., Brno, Czech Republic) densitometer. Then, the resulting suspension was diluted with saline to the desired concentration for infection. One milligram of culture was equivalent to about 10^6^ colony-forming units (CFU) as determined from a sample of suspension plated to confirm the number of viable bacilli. 

Mice were infected i.v. by injection of about 10^6^ CFU or 0.2 mL of the above suspension via the lateral caudal vein. The mice were weighed and clinically examined daily. Every second day, beginning from day 7 after inoculation, a random mouse from the untreated (**U**) group was euthanized and investigated on autopsy in order to reveal macroscopic foci of infection. Starting from day 7 after inoculation, multiple submiliary (<1 mm in diameter) and few miliary foci of inflammation were observed in inner organs, mostly in the lungs and liver. The mice developed severe infection by 11 to 13 days and lost weight during TB progression. The animals in the control group were given i.v. saline.

Treatment and survival analysis. As listed above, treatment in all infected groups, except group U, started from the 13th day after inoculation. Every day, the dead animals were autopsied with visual evaluation of the degree of macroscopic lung damage (DLD) [37,38,39], labeled and placed in 10% buffered formalin solution. All mice were maintained until death of 97% animals in control U group. All animals that survived to the 54th day of experiment were euthanized and explored for severity of lung involvement, lung weight, and index of lung bacilli insemination (lgCFU), calculated as

lgCFU = lg(CFU × L_m_ × 10^6^), (6)

where L_m_ is lung mass in grams. The colony-forming unit (CFU) was determined as follows: the lungs were aseptically removed and weighed, and 0.1 g of each lung was separately pooled and homogenized in sterile saline. Serial dilutions of the homogenates were prepared in sterile saline. The fifth dilution was inoculated onto duplicate set of slants of Lőwenstein–Jensen medium, and incubated for 21 to 28 days at 37 °C. After appearance of visible growth, the isolated colonies were counted [40]. Survival curves in all groups were plotted and compared by statistical methods described below.

Pathomorphological studies. The samples of lung, liver, spleen, and brain were taken from mice of all groups. The samples were fixed in 10% buffered formalin. Paraffin slices were prepared and stained as described above. Optical microscopy of sections was performed with a Karl Zeiss Primo Star microscope and a Leica DC fluorescence microscope (Leica Microsystems GmbH, Wetzlar, Germany).

#### 2.3.8. Statistical Analysis

The results are expressed as means ± standard deviation (SD). The intergroup variation was measured by one-way analysis of variance (ANOVA) followed by Fisher’s least significant difference test. The survival curves in pathophysiological experiments were analyzed by comparing multiple samples with log-rank Mantel–Cox test and Cox’ F-criterion [41]. Statistical significance of the results everywhere was calculated as *p* < 0.05 or better.

## 3. Results

### 3.1. Particle Preparation and PCZ Loading

**Figure 1:**
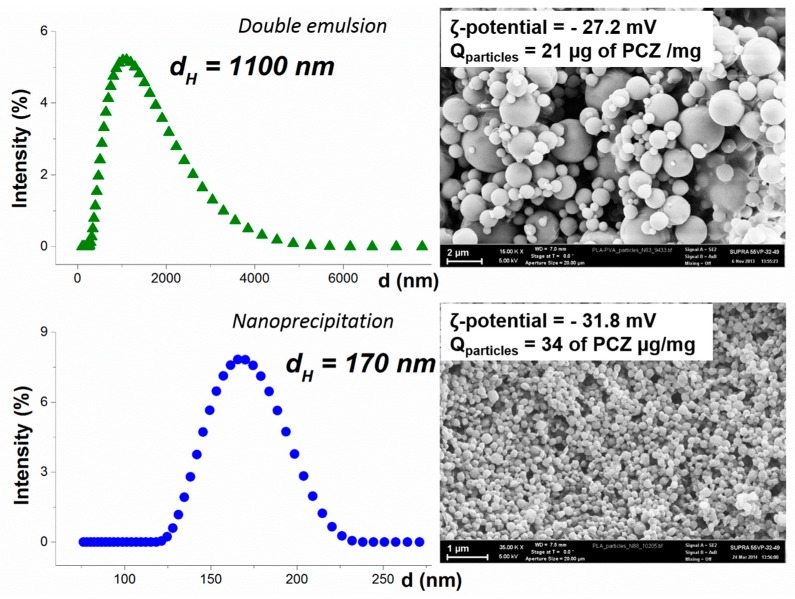
Characteristics of perchlozone loaded poly(lactic acid) (PLA) particles used for treatment of animals. Above: microparticles obtained by double emulsion method; below: nanoparticles obtained by nanoprecipitation.

Perchlozone encapsulation into PLA-based micro- and nanoparticles was performed using double emulsion evaporation and nanoprecipitation methods, respectively. The mean hydrodynamic diameter of microparticles was 1100 nm and the particle size distribution was relatively broad (Figure 1). The nanoparticles showed mean hydrodynamic diameter of 170 nm (Figure 1). The mean sizes of dry particles (scanning electron microscopy images) and particle suspensions (dynamic light scattering) matched with each other (Figure 1). The mean ζ-potential values of microparticles and nanoparticles with perchlozone were slightly negative. This was caused by the terminal carboxylic groups of PLA on the surface of the particles.

Perchlozone was successfully loaded into nano- and microparticles. Drug entrapment levels increased from 8 to 16 mg/g of particles by applying lecithin as stabilizer. Lecithin has a low hydrophile/lipophile balance (HLB) number and it formed fine water-in-oil emulsion during the particle preparation process. Overall, the perchlozone encapsulation efficacy (relative to the theoretical value) was 11% and 32% for microparticles and nanoparticles, respectively. The amount of drug entrapped was 16 mg/g of microparticles and 50 mg/g of nanoparticles. 

Camel IgG was successfully attached to both micro- and nanoparticles. The amounts of bound protein were 1.2 and 2.0 mg/g of micro- and nanoparticles, respectively.

The IR spectra of the microparticles and nanoparticles loaded with perchlozone showed strong C=S bond vibration band at 1370 cm^−1^. This reflected the presence of perchlozone in the particles. The study of particles with XRD did not reveal any perchlozone crystalline phase, but an amorphous halo of the PLA was detected. 

### 3.2. In Vitro Particle Degradation and Perchlozone Release 

**Figure 2:**
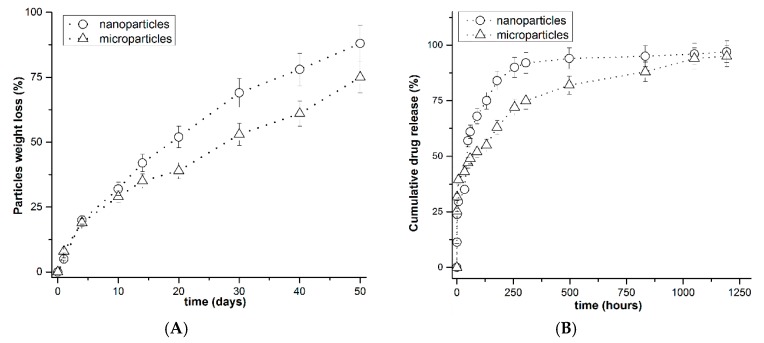
Kinetic curves of PLA particle weight loss (**A**) and perchlozone release (**B**). Conditions: 0.01 M phosphate-buffered saline (PBS), pH 4.5 containing 0.1% SDS and 0.5 mg/mL papain. The standard deviation is given as error bars.

Degradation of the particles was studied in vitro in 0.01 M PBS (pH 4.5) with 0.1% SDS and 0.5 mg/mL papain. The weight loss of the particles over 50 days was 75% and 88% for microparticles and nanoparticles, respectively (Figure 2A). The more rapid degradation of nanoparticles may be due to the greater surface area of the nanoparticles. 

Perchlozone release studies revealed a burst release of about 30% of the encapsulated drug (Figure 2B). The perchlozone release rate was faster from nanoparticles than from microparticles. Fitting of perchlozone release data using the Korsmeyer–Peppas model revealed that the main mechanism of drug release was diffusion (see Figure S1). This is in line with the fact that perchlozone release from the particles was faster than the degradation of the particles. 

### 3.3. Particle Phagocytosis In Vivo

Both PLA-based micro- and nanoparticles were modified with two fluorescent protein/Cy3 conjugates: BSA–Cy3 and camel IgG–Cy3. The labeled particles were injected intraperitoneally into the mice and the peritoneal fluid was then taken and analyzed. 

**Figure 3:**
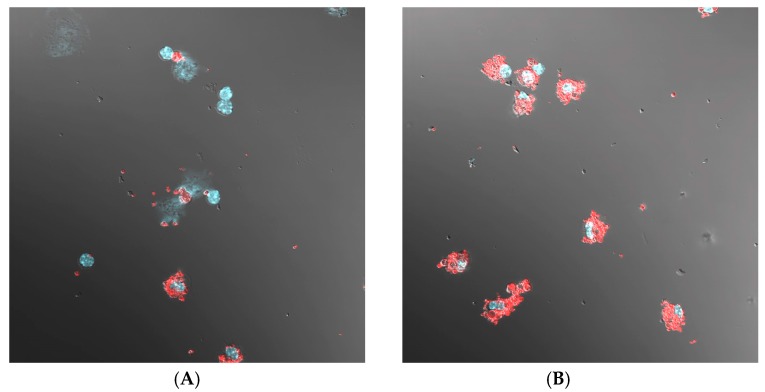
Confocal microscopy images of 4′,6-diamidino-2-phenylindole (DAPI)-stained macrophages, derived from the peritoneal cavity of the mice, with (**A**) PLA microparticles modified by bovine serum albumin (BSA)–Cy3; (**B**): PLA microparticles modified by camel immunoglobulin G (IgG)–Cy3.

**Figure 4:**
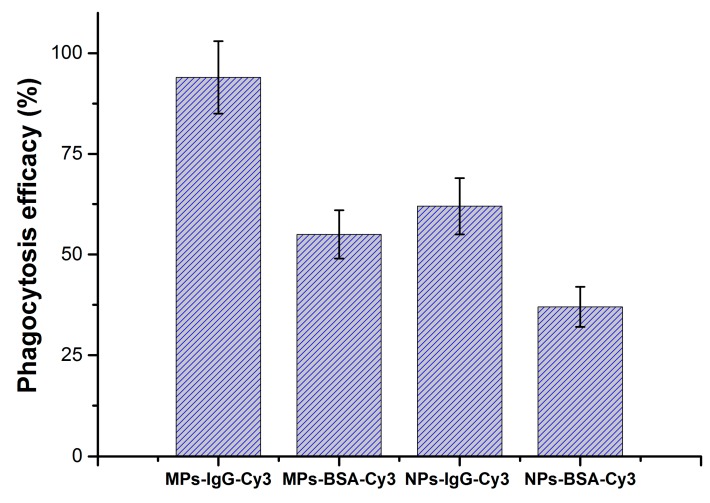
The effect of PLA particle size and IgG attachment on the percentage of macrophages derived from the peritoneal cavity of mice with internalized particles compared to the total number of macrophages.

The labeled particles were found mostly inside of the phagocytic cells (Figure 3). Analysis of the slides with confocal microscopy showed greater cellular uptake of IgG–Cy3-modified microparticles (Figure 3B) than BSA–Cy3-modified microparticles (Figure 3A). The phagocytic delivery was evaluated by analyzing the fraction of macrophages with phagocytosed micro- and nanoparticles. The data revealed that (1) attachment of IgG enhanced phagocytosis of both micro- and nanoparticles and (2) the phagocytosis of IgG-containing microparticles was more effective than phagocytosis of nanoparticles (Figure 4). Overall, it could be concluded that internalization of all types of particles in the mouse peritoneum was quite effective.

### 3.4. Survival Data and Severity of TB Involvement among Survived Mice

**Figure 5:**
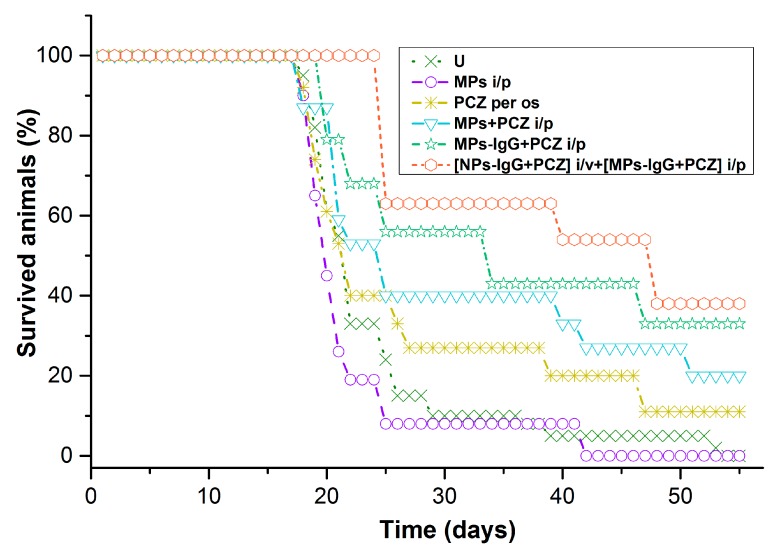
Survival of the multidrug resistance tuberculosis (MDR-TB)-infected mice after various treatments. Time “0” is the starting point of MDR-TB inoculation; the treatment started on the 13th day of infection. **Group U:** untreated control animals; saline was given orally daily. **Group MPs i.p.:** mice were treated with drug-free PLA microparticles once intraperitoneally (i.p.) **Group PCZ per os:** perchlozone was given daily orally as saline solution. **Group MPs + PCZ i.p.:** mice were treated with perchlozone loaded PLA microparticles, once i/p; **Group MPs-IgG + PCZ i/p:** the mice were injected once i.p. with perchlozone loaded PLA microparticles with camel IgG. **Group [NPs-IgG + PCZ] i.v. + [MPs-IgG + PCZ] i.p.**: mice were treated with perchlozone loaded and IgG covered PLA nanoparticles, once i.v. and, after one week, an additional i.p. injection of perchlozone-loaded PLA microparticles was delivered. For peculiarities and doses, see Table 1.

Mean longevity (Figure 5) in the untreated mouse group (**U**) was 23.7 ± 1.3 days and the survival time of the mice with oral perchlozone treatment was 28.5 ± 3.5 days. The survival time of animals treated with unloaded particles (22.4 ± 2.0 days) did not differ from the controls. However, single i.p. injection of perchlozone-loaded microparticles extended survival of animals to 30.7 ± 3.6 days (*р* < 0.05, compared with **U**). Single i.p. injection of perchlozone loaded and IgG-opsonized microparticles resulted even longer survival time (33.2 ± 3.0 days; *p* < 0.05, compared with U). 

The longest mean survival time of 37.6 ± 5.0 days was seen (Figure 5) in mice with combined treatment including i.v. injection of IgG-opsonized nanoparticles with entrapped perchlozone and i.p. injection of IgG-opsonized microparticles, also with entrapped perchlozone. It should be noted here that total doses of perchlozone were much lower in the particle injection groups (44 mg/kg) than during the daily oral dosing group (600 mg/kg). For detailed dosage description, see Table 1.

**Figure 6:**
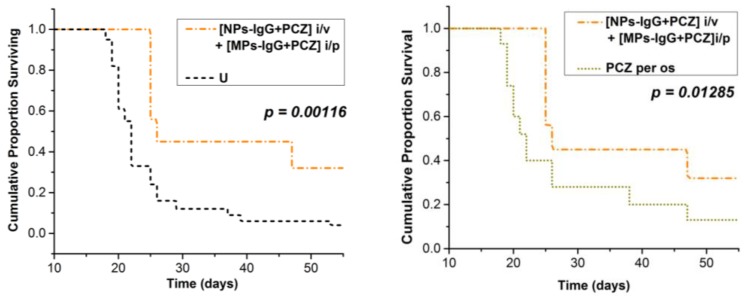
Log-rank Mantel–Cox survival curves comparison: (**A**) **[NPs-IgG + PCZ] i.v. + [MPs-IgG + PCZ] i.p.** group versus **U** group; (**B**) **[NPs-IgG + PCZ] i.v. + [MPs-IgG + PCZ] i.p.** group versus **PCZ per os** group (see Table 1 for group descriptions).

Survival curves were analyzed with a log-rank Mantel–Cox test. Extended survival of mice was seen in the group that received perchlozone as intravenous nanoparticle injections and intraperitoneal injection of perchlozone-loaded microparticles one week later ([NPs-IgG + PCZ] i.v. + [MPs-IgG + PCZ] i.p.) (Figure 6). This group showed significantly greater longevity of mice compared with untreated animals (Figure 6A), and those treated with oral perchlozone treatment (Figure 6B).

Survival rate in the **PCZ per os** group was higher than in the untreated control group (**U**). However, comparison of lung damage degree, lung weight, and lgCFU insemination indexes of all animals survived which until the 54th day of experiment showed less damage and tendency to less insemination in the joint cohort of survived animals from **MPs + PCZ i.p.** versus those of the **PCZ per os** group (Table 2). One survived animal from **[NPs-IgG + PCZ] i.v. + [MPs-IgG + PCZ] i.p.** group even gave a negative result in the CFU inoculation test.

### 3.5. Concentration of Encapsulated Fluorescent Label and Perchlozone in the Organs of Experimental Mice 

**Figure 7:**
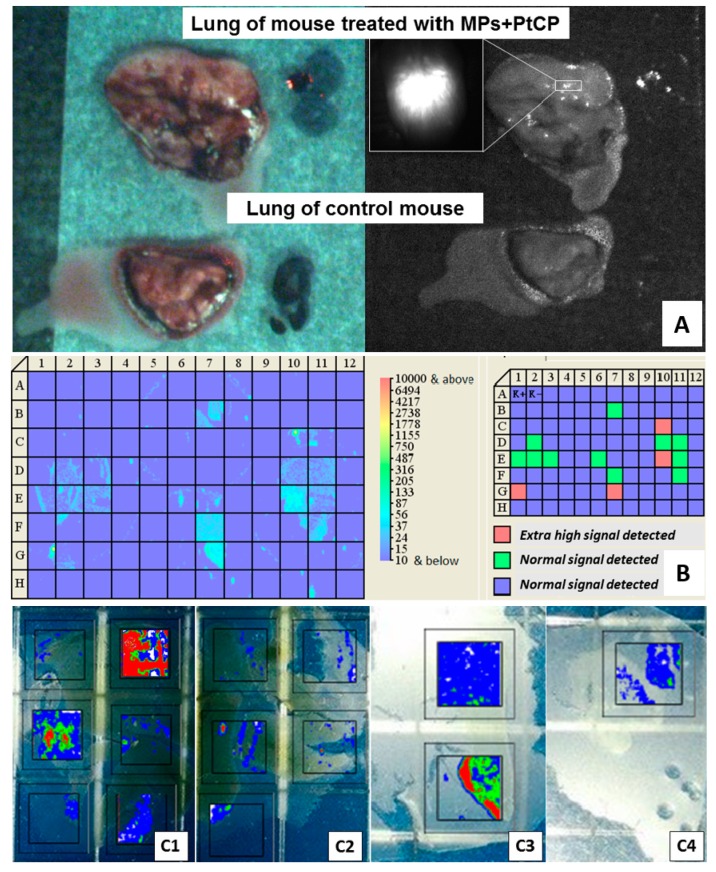
The results of phosphorescent platinum coproporphyrin (PtCP) luminescence on the surface (**A**) and within the slice of the lung (**B**) of mice on the seventh day after i.p. injection of particles (group **MPs-IgG + PCZ i.p**.) obtained with a sensitive laser fluorometric scanner diagram; (**C**) the comparison of tissue slices (after 50 days) luminescence of different organs: (**C1**) lung, group **MPs-IgG + PCZ i.p.**; (**C2**) lung, group **U**; (**C3**) liver **MPs-IgG + PCZ i.p.**; (**C4**) brain.

The accumulation of particles in the lung was imaged by detection of PtCP label luminescence, which was encapsulated into the particles together with perchlozone (Figure 7). The luminescence of encapsulated PtCP was visualized both on the surface (Figure 7A) and within the slices (Figure 7B) of the derived lung of mouse from the **MPs + PCZ i.p.** group. The greater luminescence was seen after treatment of MDR-TB-infected lung (Figure 7C1) as compared to untreated lung (Figure 7C2) and luminescence in the liver and brain (Figure 7C3 and C4, correspondingly). Quantification of PtCP concentration in different organs of the mice was also performed (Table 3). The results showed significant particle accumulation within foci of infection in the lung and liver in the animals treated with microparticles. Traces of signal at slices of untreated mice are commonly caused by presence of natural Zn-containing proteins [42]. Greater particle accumulation was detected in the case of microparticles with camel IgG (Table 3). Pathomorphologically, the areas of high signal contained foci of TB inflammation with mononuclear cell infiltration (see Table 2).

Detection of perchlozone concentrations with LC–MS revealed higher concentrations of the drug in the lungs after intraperitoneal injection of perchlozone microparticles with camel IgG as compared to the steady-state concentration during daily multiple dose delivery of perchlozone orally (Table 4). 

### 3.6. Pathomorphological Data 

**Figure 8:**
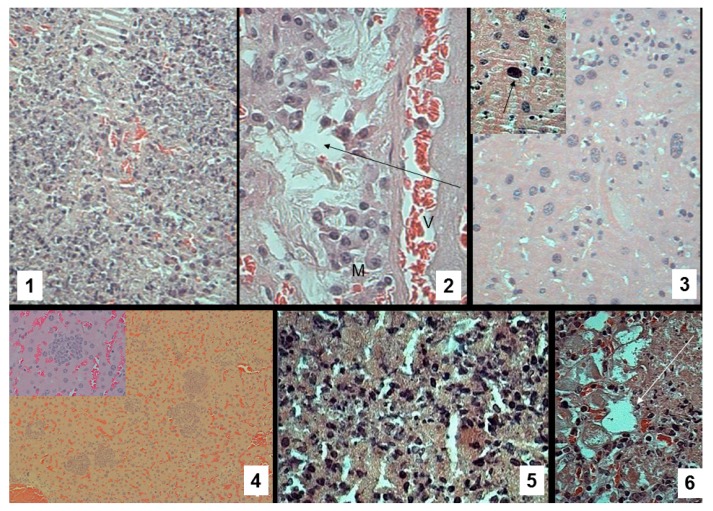
Pathomorphological data (hematoxylin and eosin (H&E) stain): **1**—lung, 20th day of TB, degree of macroscopic drug damage (DLD) = 3.75, group **MPs + PCZ i.p.**, objective 40×; **2**—same animal, lung, 80×, near blood vessel (V) collection of macrophages *(M)* and presumable deposit of particles (arrow); **3**—liver, 18th day of TB, DLD = 3.25, group **PCZ per os**, 80×, typical TB involvement; on top left insertion, same animal, nuclear changes (arrow) typical for PCZ toxic effect; **4**—liver, 18th day of TB, DLD = 3.75, group **MPs-IgG + PCZ i.p.**, 10× (insertion 40×), granulomas, but no degenerating cells were found; **5** and **6**—same animal as **1** and **2**, lung TB, **5**—objective 80×; **6**—deposit of particles (arrow) in TB focus.

We succeeded in revealing microscopic pictures typical for experimental TB in the lungs and liver (Figure 8) in all infected animals. In the group that received perchlozone orally, we observed regular nuclear hyperchromatosis (Figure 8(3)), related to the toxic effects of perchlozone [5,7]. The animals that received perchlozone within microparticles (**MPs + PCZ i.p.** group) demonstrated no such toxic actions (Figure 8(1,2,4)). In Figure 8(4), at low magnification, many “standard” granulomas were visible, but at magnification (40×), no hyperchromic degenerating cells were found in the granuloma. Some polymorphism of macrophages was observed, indirectly indicating their activity. The tendency to remedial effect of the treatment with perchlozone-loaded particles was evident. In the lungs of the treated animals, we succeeded in detecting considerable amounts of exogenous light eosinophilic non-structured material in peribronchial and perivascular spaces, which were considered to be degraded particles (Figure 8(2,6)).

## 4. Discussion

Despite previous scientific and medical efforts, tuberculosis still remains a severe disease, which kills a lot of people every day [23]. The antibiotics that are used to treat tuberculosis are often quite toxic and ineffective against resistant strains. A novel drug against the resistant forms of tuberculosis, 4-thioureidoiminomethylpyridinium perchlorate (perchlozone^©^), also has serious adverse effects [5]. Therefore, we aimed to improve the delivery of perchlozone in order to reduce the side-effects. 

Different particles were tested for reduction of anti-tuberculosis drug toxicity and for targeting drugs into macrophages [18,43]. Nevertheless, the particles in these studies were delivered via a pulmonary route to target macrophages in the lung. Most studies dealt with encapsulation of old drugs like rifampicin [15], ofloxacin [18], and dexamethasone [43]. We report here the properties of encapsulated forms of a new drug with activity against resistant tuberculosis.

The idea of our study was to apply the non-inflamed macrophages from intraperitoneal fluid as delivery vehicles of the novel drug perchlozone, to treat MDR-TB. We propose that PLA-based particles, which were internalized by peritoneal macrophages, move to the inflammation site in the lung via chemotaxis. In this way, the systemic distribution of perchlozone could be reduced and efficacy of the drug could be improved. 

Perchlozone was successfully loaded into PLA micro- and nanoparticles using modified double emulsion and nanoprecipitation methods, respectively. Perchlozone is a quite a hydrophilic drug, and its encapsulation efficiency into microparticles via emulsification approach was initially low, presumably due to the instability of the water-in-oil emulsion. The loading was improved with surfactant with low hydrophilic/lipophilic balance number (lecithin with HLB = 4). Successful entrapment of perchlozone was proven photometrically and with FTIR spectroscopy. Importantly, XRD studies did not show any separate perchlozone phases in the particles, suggesting that the drug was homogenously dispersed into the PLA matrix. The encapsulation of perchlozone via nanoprecipitation resulted in slightly greater efficacy, which could be explained by rapid PLA phase separation and trapping of drug molecules inside the precipitated particles. As far as we know, this is the first study that combines micro- and nanoparticles for treatment of tuberculosis.

The particle degradation and perchlozone release were studied in a medium that mimics the environment in the macrophages. The obtained results (Figure 2) suggest that particles could be stable in the phagocytes for 10 days, which is adequate time for the transfer of macrophages to the inflammation sites in the lungs. In general, based on the results, we propose that a substantial drug quantity is delivered within macrophages to the foci of infection. 

Camel IgG is known to be a good therapeutic agent itself [44,45]. Here, it was applied for macrophage targeting, but its own therapeutic activity against tuberculosis is also possible. Both micro- and nanoparticles were successfully modified by camel IgG via activated ester approach, and the conjugated camel IgG induced opsonization and activated the phagocytosis of the particles (Figure 4). Not surprisingly, microparticles were phagocytosed more extensively than the nanoparticules (Figure 4). It was previously shown that microparticles are better recognized by macrophages than nanosized structures [46]. 

The experiments with infected mice showed that application of particulate formulations of perchlozone, especially IgG-modified particles, were more effective (animal survival improved) at clearly lower total doses than oral percholozone. A lower degree of lung damage in survived mice was also seen in mice that received injected perchlozone particles. It is noteworthy that the total dose of perchlozone in the injected particles was 44 mg/kg (Table 1), only 7.3% of the dose that was delivered orally. The data suggest that particle-encapsulated perchlozone is delivered to the lungs within the macrophages. 

The results were obtained with the application of outbred mice to mimic the genetic diversity in the human population in terms of immune responses to mycobacterial infection and drug pharmacokinetics. Despite the fact that many interesting results were obtained with the application of inbred mice [47], the application of outbred animals allowed us to test the targeting capabilities of macrophages, which do not have the same genetic defects as in the inbred mice [35]. However, one could expect even better survival with C57Bl/6 mice [28], and we are going to test this further.

Biodistribution of microparticles and perchlozone cargo was studied using fluorescent labeling (Figure 7, Table 3) and LC–MS analysis of tissue slices, respectively. The PtCP label was detected in the infected lungs suggesting particle delivery, possibly via macrophage chemotaxis. This is consistent with the pathomorphological data. Microparticles do not diffuse from the peritoneal cavity to the blood stream as such, but tend to accumulate there [48], which also supports the notion of macrophage-mediated transport. The LC–MS data showed the highest concentrations of perchlozone in the lungs as compared with the other organs, proving effective drug delivery to the lungs. It should also be noted that the microparticles (possibly with IgG) are suitable for intraperitoneal injections, because they can be internalized by macrophages for further chemotaxis [49,50]. However, the resolution of images (Figure 7 and Figure 8(6)) allows us to make only preliminary conclusions on the particles’ pharmacokinetics, and further studies are required to obtain more detailed information.

The pathomorphological data comparison (Figure 8) with the evidence on perchlozone concentrations in the organs of experimental animals (Table 4) allows the conclusion that oral administration of drug results in high drug concentration in the liver. This causes the hepatotoxicity of the drug. Intravenously administered nanoparticles seem to also deliver drugs to the liver (Table 4). However, after nanoparticle administration, the drug may not be fully released in the liver and its distribution within the liver may differ from the distribution of orally administered perchlozone. The best therapeutic effect with minimal toxicity (Figure 8(4)) was seen after application of perchlozone-loaded microparticles intraperitoneally. This favorable effect and minimal toxicity was probably due to macrophage-mediated transport to the foci of inflammation in the lungs, avoidance of the first pass metabolism, and differences of drug distribution in the liver (among cell types, encapsulated vs. free drug). We also need to mention that possible hepatotoxic effects of perchlozone alone after i.p. and i.v. administration were not tested. 

## 5. Conclusions

The PLA-based micro- and nanoparticles were loaded with anti-tuberculosis drug perchlozone, and successfully applied for treatment of MDR-TB in mice. The best survival was observed in animals that were treated with intravenous IgG-modified nanoparticles and intraperitoneal IgG-modified microparticles, both loaded with perchlozone. Targeted particle delivery to the foci of infection in tuberculosis mice was seen. The observed greater longevity of treated experimental mice could be attributed to the application of PLA-based perchlozone-loaded microparticles, modified by camel IgG.

Further studies are needed to understand the pathways and mechanisms of particle distribution in the body. Nevertheless, the applied treatment could be proposed for MDR-TB so as to decrease the drug toxicity.

## Figures and Tables

**Table 1:** The description of groups of mice involved in the experiment. Abbreviations: p/o—per os; i.p.—intraperitoneally; i.v.—intravenously; PCZ—perchlozone; IgG—immunoglobulin; TB—tuberculosis.

Abbreviation	Description of Animals/Periodicity of Treatment/Type of Particles	Means of Treatment	Suspension Volume/Concentration/Medium	Dose	Number of Animals
**N**—non-treated	healthy controls/no treatment/no particles	none	none	none	15
**U**—untreated	TB-infected mice/daily/no particles	p/o	0.2 mL of pure saline	none	33
**PCZ per os**—perchlozone per os	TB-infected mice/daily/no particles	p/o	0.5 mL of drug solution 0.6 mg/mL in saline	single dose = 15 mg/kg total dose for survived mice = 600 mg/kg	15
**MPs i.p.**—microparticles introperitoneally	TB-infected mice/once/microparticles	i.p.	0.5 mL/10 wt.%/saline	none	11
**MPs + PCZ i.p.**—microparticles + perchlozone intraperitoneally	TB-infected mice/once/microparticles	i.p.	0.5 mL/10 wt.%/saline	single dose and total dose = 41 mg/kg	15
**MPs-IgG + PCZ i.p.**—microparticles modified with IgG + perchlozone intraperitoneally	TB-infected mice/once/microparticles modified with camel IgG	i.p.	0.5 mL/10 wt.%/saline	single and total dose drug = 41 mg/kg. IgG = 3 mg/kg.	11
**[NPs-IgG + PCZ] i.v. + [MPs-IgG + PCZ] i.p.**—nanoparticles loaded with perchlozone and modified with camel IgG intravenously and microparticles loaded with perchlozone and modified with camel IgG intraperitoneally	TB-infected mice/once intravenously by nanoparticles loaded with perchlozone and modified with camel IgG intravenously and 1 week later microparticles loaded with perchlozone and modified with camel IgG intraperitoneally/nano- and microparticles	i.v. + i.p.	i.v.: 0.2 mL/10 wt.%/saline i.p.: 0.5 mL/10 wt.%/saline	drug dose: i.v. = 3 mg/kg, i.p. = 41 mg/kg. Total dose of drug = 44 mg/kg immobilized IgG doses: i.v. = 2 mg/kg, i.p. = 3 mg/kg Total dose of IgG = 5 mg/kg	9

**Table 2:** Degree of lung involvement and insemination in survived mice. Abbreviations: L_m_—mass of the lung; DLD—degree of macroscopic drug damage (see Section 2); CFU—colony-forming unit; lgCFU—index of lung bacilli insemination.

Parameter	Group	
	PCZ per os	MPs + PCZ i/p
L_m_, g	0.59 ± 0.06	0.41 ± 0.05
L_m_ % of body mass	1.97 ± 0.20	1.33 ± 0.20
DLD	3.36 ± 0.27	2.50 ± 0.17
lg CFU	2.08 ± 0.60	1.21 ± 0.44

**Table 3:** Quantitative estimation of total organ slice luminescence (counts per standardized section square).

Organ	Group		
	U Mouse, 21st Day of TB, DLD = 4.0	MPs + PCZ i.p. Mouse, 20th Day of TB, 7th Day after Injection DLD = 3.75	MPs-IgG + PCZ i.p. Mouse, 21st Day of TB, 8th Day after Injection DLD = 3.75
Liver	249 ± 27	1314 ± 144	2004 ± 220
Lung	287 ± 32	1536 ± 169	3865 ± 425
Spleen	69 ± 8	238 ± 26	538 ± 59
Brain	30 ± 3	155 ± 17	188 ± 21

**Table 4:** Quantitation of perchlozone in the mouse tissues with LC–MS.

Organ	Group		
	PCZ Concentration in the Slices of PCZ per os Mouse, 20th Day of TB, 7th Day after Injection (µg/slice)	PCZ Concentration in the Slices of MPs-IgG + PCZ i.p. Mouse, 21st Day of TB, 8th Day after Injection (µg/slice)	PCZ Concentration in the Slices of [NPs-IgG + PCZ] i.v. + [MPs-IgG + PCZ] i.p. Mouse, 21st Day of TB, 8th Day after Injection (µg/slice)
Liver	335.2 ± 52.8	154.5 ± 23.2	297.2 ± 44.5
Lung	132.0 ± 19.8	283.0 ± 42.5	307.8 ± 46.2
Spleen	36.9 ± 5.5	59.9 ± 9.0	55.5 ± 8.3
Brain	1.2 ± 0.2	<0.03	<0.03

## Supplementary Materials

The following is available online at http://www.mdpi.com/1999-4923/11/1/2/s1: Figure S1: The linearization of perchlozone release plots.

## Abbreviations

TB	tuberculosis
HIV	human immunodeficiency virus
MDR-TB	multiple drug resistant *M. tuberculosis*
PLA	poly(d,l-lactic acid)
ICG	indocyanine green
IgG	immunoglobulin G
PtCP	platinum(II)-coproporphyrin
CFU	colony-forming units
LC–MS	liquid chromatography/mass spectrometry
