# Supplementary material for: Enhanced Delivery of 4-Thioureidoiminomethylpyridinium Perchlorate in Tuberculosis Models with IgG Functionalized Poly(Lactic Acid)-Based Particles
Source: Pharmaceutics. 2018 Dec 21;11(1):2. doi: 10.3390/pharmaceutics11010002 (PMC6359407; doi:10.3390/pharmaceutics11010002)
Supplement: Supplementary file 1 [file pharmaceutics-11-00002-s001.pdf]

# Supplementary Materials: Enhanced Delivery of 4-Thioureidoiminomethylpyridinium Perchlorate in Tuberculosis Models with IgG Functionalized Poly(Lactic Acid)-Based Particles

**Leonid Churilov, Viktor Korzhikov-Vlakh, Ekaterina Sinitsyna, Dmitrii Polyakov, Oleg Darashkevich, Mikhail Poida, Galina Platonova, Tatiana Vinogradova, Vladimir Utekhin, Natalia Zabolotnykh, Vsevolod Zinserling, Peter Yablonsky, Arto Urtti and Tatiana Tennikova ***

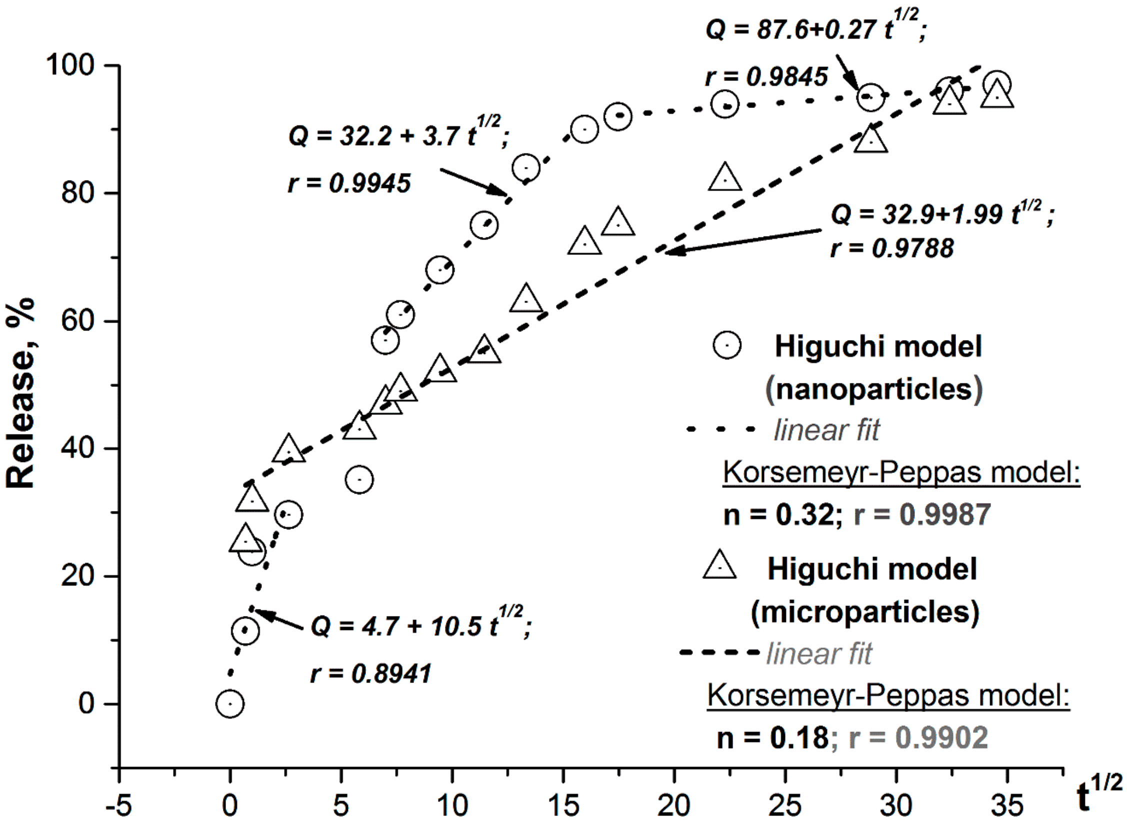


**Figure S1.** The linearization of perchlozone release plots.
